# Trajectory of worst pain within the first two weeks following pelvic and sacral tumor surgery and long-term outcome: a pilot observational prospective cohort study

**DOI:** 10.1186/s12871-023-02033-z

**Published:** 2023-03-09

**Authors:** Qingfen Zhang, Yaqing Wu, Shenda Hong, Yi Feng

**Affiliations:** 1 Department of Anesthesiology, Peking University People’s Hospital, Beijing, 100044 China; 2 National Institute of Health Data Science, Peking University, Beijing, 100191 China; 3 Institute of Medical Technology, Peking University Health Science Center, Beijing, 100191 China

**Keywords:** Sacrectomy, Pelvic resection, Acute postsurgical pain, Persistent postsurgical pain, Pain trajectory, Opioids

## Abstract

**Background:**

Pain management after pelvic and sacral tumor surgery is challenging and requires a multidisciplinary and multimodal approach. Few data on postoperative pain trajectories have been reported after pelvic and sacral tumor surgery. The aim of this pilot study was to determine pain trajectories within the first 2 weeks after surgery and explore the impact on long-term pain outcomes.

**Methods:**

Patients scheduled for pelvic and sacral tumor surgery were prospectively recruited. Worst/average pain scores were evaluated postoperatively using questions adapted from the Revised American Pain Society Patient Outcome Questionnaire (APS-POQ-R) until pain resolution was reached or up to 6 months after surgery. Pain trajectories over the first 2 weeks were compared using the k-means clustering algorithm. Whether pain trajectories were associated with long-term pain resolution and opioid cessation was assessed using Cox regression analysis.

**Results:**

A total of 59 patients were included. Two distinct groups of trajectories for worst and average pain scores over the first 2 weeks were generated. The median pain duration in the high vs low pain group was 120.0 (95% CI [25.0, 215.0]) days vs 60.0 (95% CI [38.6, 81.4]) days (log rank *p* = 0.037). The median time to opioid cessation in the high vs low pain group was 60.0 (95% CI [30.0, 90.0]) days vs 7.0 (95% CI [4.7, 9.3]) days (log rank *p* < 0.001). After adjusting for patient and surgical factors, the high pain group was independently associated with prolonged opioid cessation (hazard ratio [HR] 2.423, 95% CI [1.254, 4.681], *p* = 0.008) but not pain resolution (HR 1.557, 95% CI [0.748, 3.243], *p* = 0.237).

**Conclusions:**

Postoperative pain is a significant problem among patients undergoing pelvic and sacral tumor surgery. High pain trajectories during the first 2 weeks after surgery were associated with delayed opioid cessation. Research is needed to explore interventions targeting pain trajectories and long-term pain outcomes.

**Trial registration:**

The trial was registered at ClinicalTrials.gov (NCT03926858, 25/04/2019).

## Introduction

Pelvic and sacral tumors are difficult to diagnose due to their deep location and lack of typical symptom presentation at the early stage [1]. The growing tumor can cause intense mechanical and neuropathic pain by mass effect and infiltration into nearby structures. Surgery is the most important treatment [2, 3]; however, it is also challenging due to the enlarged tumor and the complicated anatomy of the pelvis and sacrum. Surgical procedures usually take hours and may be accompanied by massive hemorrhage [2, 4, 5].

Pain after pelvic and sacral tumor surgeries is a significant problem and is usually undertreated [4]. Inadequately managed acute pain can lead to physiological and psychological consequences, a prolonged hospital stay and an increased financial burden. In addition, it may increase the risk of developing chronic postsurgical pain [6], thus leading to a poorer health-related quality of life [7].

To date, no study has comprehensively examined postoperative pain after pelvic and sacral tumor surgeries. We are unclear how severe acute pain can be and the extent to which severe acute pain may impact long-term outcomes such as the time to pain resolution and opioid cessation. Persistent postsurgical pain after surgery is a common issue [8], and the overall incidence is approximately 20–30% for all types of surgery [9]. Whether chronic pain is a greater concern for this specific group is unclear.

In this pilot study, we examined pain trajectories in patients who underwent sacral and pelvic tumor surgery over the first 6 months after surgery. We proposed pain trajectories within the first 2 weeks following surgery associated with long-term pain resolution and opioid cessation.

## Methods

This analysis was a substudy of a multicenter prospective study of pain and health-related quality of life after surgery and was registered at ClinicalTrials.gov (NCT03926858, 25/04/2019). This study was approved by the Ethics Committee of Peking University People’s Hospital (2018PHB229–02) and conducted in compliance with the Declaration of Helsinki. Enrollment was initiated after registration and written informed consent was obtained from all participants. This study was reported following Strengthening the Reporting of Observational Studies in Epidemiology guidelines.

### Participants

Hospitalized patients aged 18 years or older with pelvic or sacral tumors who were scheduled for sacrectomy or pelvic resection were screened from the operation list. Exclusion criteria were existing diagnoses of major psychiatric disorders, surgery cancellation, an anticipated postoperative intubation longer than 24 hours, inability to read or write, or inability to give informed consent. All patients underwent surgery under general anesthesia at Peking University People’s Hospital between January 2020 and July 2021.

### Study protocol

All patients were consecutively recruited on the day before surgery. After written informed consent was obtained, baseline questionnaires were completed. Follow-ups after surgery for pain evaluation were performed on postoperative days (POD) 1, 3, 7, 14, 21, and 30 and monthly thereafter until pain resolution was reached or up to 6 months after surgery; the sessions were conducted by face-to-face interviews during the hospital stay or telephone interviews after discharge. Loss to follow-up was defined as the patient not being contacted during two consecutive follow-ups.

A standard perioperative pain management protocol was performed. Multimodal analgesia during surgery included the following: 1) corticosteroids, such as intravenous injection of methylprednisolone 40–80 mg before induction; 2) continuous infusion of dexmedetomidine at a rate of 0.4–0.6 μg/kg/h until incision closure; 3) short-acting opioids, including intermittent intravenous injection of sufentanil with a total dose of 0.5–1.0 μg/kg and continuous infusion of remifentanil 0.1–0.2 μg/kg/min until the end of surgery; and 4) flurbiprofen 100 mg or parecoxib 40 mg intravenously administered before the end of surgery when no contraindication presented. At the end of surgery, patient-controlled intravenous analgesia (PCIA) with sufentanil was provided to each patient for at least 72 hours. The PCIA device was initially set to deliver sufentanil at a rate of 2 μg/hour (solution 1 μg/ml) and a bolus of sufentanil 3 μg on request with a lockout time of 15 minutes. Background infusion was stopped if the worst pain score was <= 3 or opioid-related side effects (such as nausea and vomiting and dizziness) were reported during follow-ups. If severe opioid-related side effects persisted despite pharmacological treatment, PCIA was stopped at the request of the patient.

In wards, nonsteroidal anti-inflammatory drugs or COX-2 inhibitors were used as needed based on the surgeons’ preference. If patients reported pain with neuropathic characteristics, such as numbness and burning, gabapentin was added. Immediate-release oxycodone (5 mg) or tramadol (100 mg) was administered orally for rescue analgesia. Oral sustained-release oxycodone (5 mg every 12 hours) or a transdermal fentanyl patch (25 μg/hour for 72 hours) was provided for persistent severe pain after cessation of PCIA. Pain consultations were held when necessary.

### Data collection

Before surgery, demographic information was collected through a patient-reported questionnaire. Preoperative anxiety and depressed mood were assessed using a 0 (not anxious or depressed) to 10 (extremely anxious or depressed) scale. The Chinese version of the Pain Catastrophizing Scale (PCS) [10] was used to evaluate rumination, magnification, and helplessness associated with pain. Preoperative chronic pain (lasting for at least 3 months) was evaluated using the Brief Pain Inventory-Short Form [11]. Preoperative health-related quality of life was evaluated using the validated Chinese version of EuroQol five-dimensional-5 levels (EQ-5D-5L) questionnaire [12]. Postoperative pain was assessed using part of the Revised American Pain Society Patient Outcome Questionnaire (APS-POQ-R) [13]. Specifically, patients reported the worst, average and lowest pain scores over the last 24 hours and the current pain scores related to the surgery at the time of interview. Pain resolution was defined as two consecutive reports of no pain, with no analgesics or pain control therapies needed.

The patients’ medical records were reviewed to obtain the Charlson Comorbidity Index and surgery and analgesia information. Data on surgical complications were obtained from medical records during the hospital stay and patient self-reports after discharge. Major complications were defined as complications of grade III or higher according to the Clavien–Dindo Classification [14]. Because of the various types and routes of opioids that were prescribed, all opioids were converted to oral morphine equivalents (MEQs) using standard formulas [15].

### Statistical analysis

Statistical analysis was performed using IBM SPSS Statistics (Version 23.0, IBM Corp, New York, USA) and the R programming language (version 4.1.1). Data are expressed using means ± (standard deviation, SD), medians (interquartile range, IQR) or frequencies (percentages). Each patient had two distinct pain trajectories for worst pain scores and average pain scores. The R package kml (K-means for longitudinal data) was used to cluster each pain trajectory category. The Euclidean distance between values at each time point was measured for clustering. Calinski–Harabasz scores were used to evaluate intergroup distinctness and intragroup variation, and the optimal number of groups corresponded to the value of k that maximized the Calinski–Harabasz scores [16]. Patients were divided into different pain trajectory groups generated by the k-means algorithm according to pain scores over the first 2 weeks after surgery.

The median time to pain resolution and opioid cessation was analyzed using Kaplan–Meier survival analysis and log rank statistics. Cox regression analysis was used to assess the correlation of the pain trajectory group with long-term outcomes, adjusting for potential patient- and surgery-related factors. Factors with *p* < 0.10 in the univariate analysis were entered into the multivariable Cox regression analysis.

Logistic regression was used to examine preoperative factors associated with the high pain trajectory group. Factors were compared between clusters by Student’s t tests, Mann–Whitney U tests, and chi-squared or Fisher’s exact tests, as appropriate. Factors with *p* < 0.10 were considered for inclusion in the final model with pain trajectory group assignment as the outcome. A 2-sided *p* value less than 0.05 was considered statistically significant.

As a substudy of a multicenter study, the sample size was determined by available data from patients enrolled in the main study, and no statistical power was calculated before analysis.

## Results

**Fig. 1:**
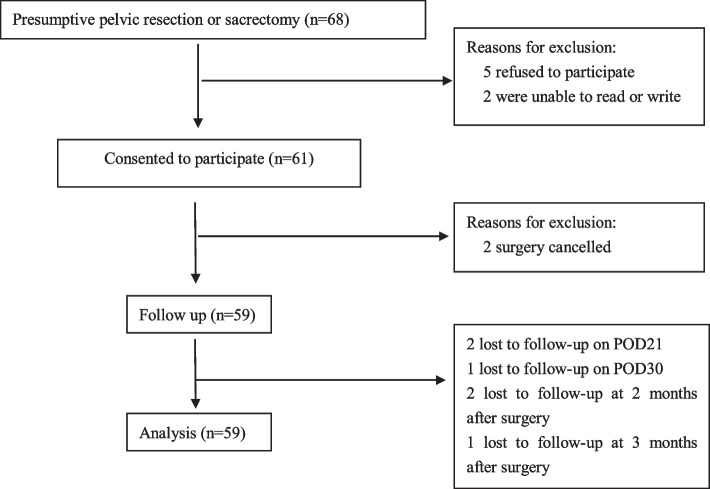
Study flowchart of the patients enrolled in the study. POD, postoperative day

A total of 68 patients were screened. Five patients refused to participate, 2 patients were excluded for inability to read or write, and 2 patients were excluded for surgery cancellation. Finally, 59 patients were included. The study flowchart is shown in Fig. 1. All patients completed preoperative questionnaires, and 53 patients (89.8%) completed follow-ups until pain resolution was reached or up to 6 months after surgery. Imputation using the last observation carried forward (LOCF) approach was used to complete the pain trajectories for patients who completed follow-ups.

### Patient characteristics

Demographic and clinical variables are summarized in Table 1. Twenty patients (33.9%) reported chronic pain before surgery, and 15 patients (25.4%) required opioids with a median daily dose (MEQ) of 10 (10, 20) mg. Persistent postoperative pain was reported by 35.8% (19/53) of the patients at 3 months and 28.3% (15/53) at 6 months. The percentage of patients who required opioids was 9.4% (5/53) at 3 months and 7.5% (4/53) at 6 months. The overall incidence of major complications after surgery was 18.6% (11/59), and all complications occurred during the hospital stay. Nine patients experienced wound healing problems and underwent debridement under local anesthesia (7 patients) or general anesthesia (2 patients). Two patients experienced cerebrospinal fluid leakage and underwent surgery under general anesthesia. No major complications were reported after discharge. No cases of tumor recurrence were reported at 6 months after surgery.

**Table 1:** Summary

Characteristics	Overall
Age (yrs)	42.6 ± 12.8
Sex, male, n (%)	30 (50.8)
BMI (kg/m^2^)	23.7 ± 3.6
Education	
Completed high school, n (%)	35 (59.3)
Less than high school, n (%)	24 (40.7)
Baseline EQ-5D score	0.94 (0.78, 1.00)
Charlson Comorbidity Index	2 (2, 5)
Chronic pain before surgery, n (%)	20 (33.9)
Opioids before surgery, n (%)	15 (25.4)
Oxycodone	13 (22.0)
Codeine	2 (3.4)
Anxiety	0 (0, 2)
Depressed mood	0 (0, 0)
PCS score	4 (0, 16)
Procedures	
Pelvic resection, n (%)	32 (54.2)
Sacrectomy, n (%)	27 (45.8)
Surgery duration (min)	216 (160, 289)
Bleeding (ml)	1000 (600, 2000)
Major complications**,** n (%)	11 (18.6)
Length of hospital stay (d)	13.0 (8.0, 18.0)
Total opioids before discharge (MEQ, mg)	605 (450, 830)

Data are shown as mean ± SD, median (IQR), or numbers (%). *Abbreviations*: *BMI* Body mass index, *PCS* Pain Catastrophizing Scale, *MEQ* Morphine equivalent

### Pain trajectory group analysis

**Fig. 2:**
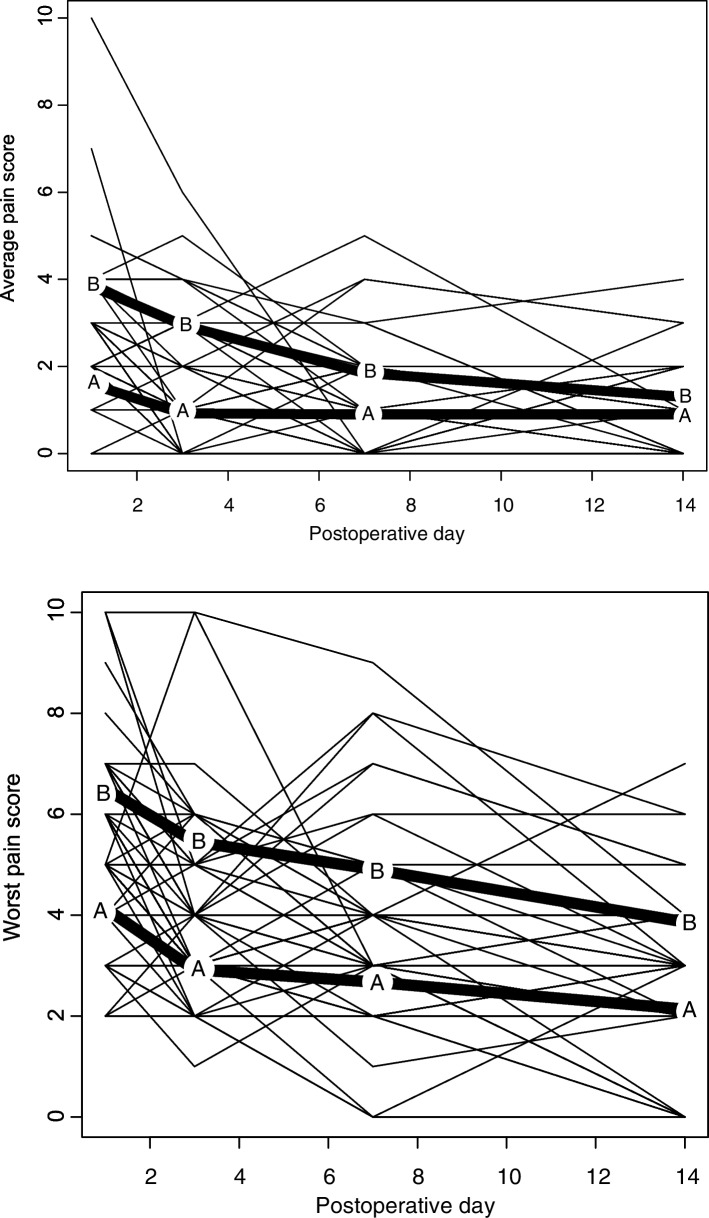
Acute pain trajectories for average pain and worst pain scores over the first two weeks after surgery. Each line describes an individual patient’s pain trajectory, and the solid Lines A and B represent the overall low and high pain groups, respectively

Each patient had two distinct pain trajectories representing the worst and average pain scores. Two groups of trajectories for both average and worst pain over the first 2 weeks were generated from k-means clustering analysis (Fig. 2). The high pain group of average pain trajectories comprised 10 patients, all of whom were also categorized into the high pain group of worst pain trajectories. Thus, further analyses were performed using groups for the worst pain trajectories, including a high pain group with 22 patients (37.3%, 22/59) and a low pain group with 37 patients (62.7%, 37/59).

After surgery, the median length of stay was 15.0 (10.8, 20.3) days in the high pain group in comparison with 10.0 (7.5, 16.0) days in the low pain group (*p* = 0.005). The rate of major complications was significantly increased to 36.5% (8/22) in the high pain group compared with 8.1% (3/37) in the low pain group (*p* = 0.013). The total median dosage of opioids administered during the hospital stay was 857 (625, 1365) mg (MEQ) in the high pain group compared with 505 (425, 632) mg (MEQ) in the low pain group (*p* < 0.001).

**Fig. 3:**
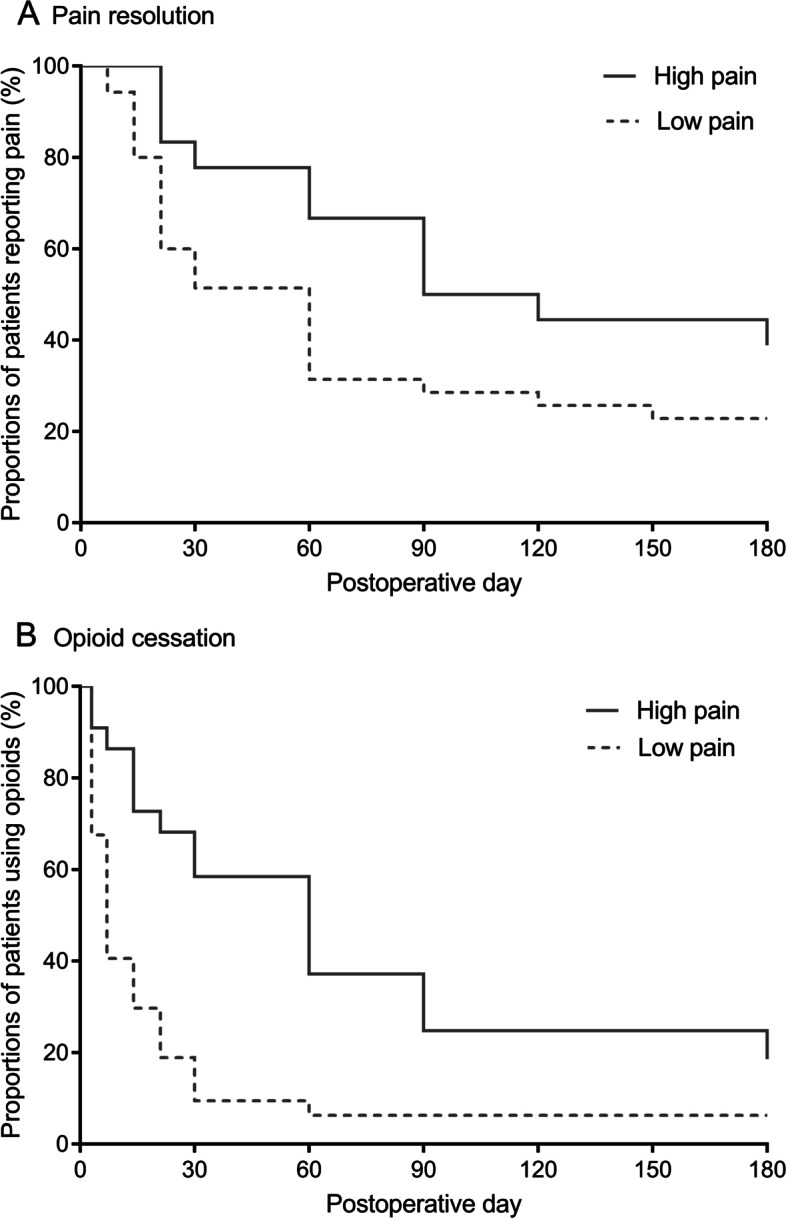
Pain outcomes comparing high and low pain groups for the worst pain trajectories. **A** Kaplan–Meier curve for pain resolution; **B** Kaplan–Meier curve for opioid cessation

Figure 3 shows the Kaplan–Meier curves stratified by high vs low pain groups. A longer duration of pain and opioid use was observed in the high pain group than in the low pain group (median 120.0 (95% CI [25.0, 215.0]) days vs 60.0 (95% CI [38.6, 81.4]) days, log rank *p* = 0.037; 60.0 (95% CI [30.0, 90.0]) days vs 7.0 (95% CI [4.7, 9.3]) days, log rank *p* < 0.001, respectively).

### Cox regression analysis

For pain resolution, pain trajectory group, age, major complications after surgery and surgery type were factors identified with *p* < 0.10 in univariate analysis. After adjustment, the pain trajectory group was not significantly associated with pain resolution (hazard ratio [HR] 1.557, 95% CI [0.748, 3.243], *p* = 0.237). For opioid cessation, pain trajectory group and surgery duration were factors identified with *p* < 0.10 in univariate analysis. The high pain group was independently associated with prolonged opioid cessation after adjustment (HR 2.423, 95% CI [1.254, 4.681], *p* = 0.008) (Table 2).

**Table 2:** Univariate and multivariable analyses of factors associated with remote pain resolution and opioid cessation after surgery

Factors	Pain resolution				Opioid cessation			
	Univariate HR (95% CI)	p	Multivariable HR (95% CI)	p	Univariate HR (95% CI)	p	Multivariable HR (95% CI)	p
Group of worst pain trajectories								
Low pain	**Referent**				**Referent**			
High pain	1.977 (0.978, 3.996)	0.058	1.557 (0.748, 3.243)	0.237	2.756 (1.471, 5.164)	0.002	2.423 (1.254, 4.681)	0.008
Age	0.976 (0.953, 1.000)	0.051	0.982 (0.957, 1.008)	0.168	0.989 (0.968, 1.011)	0.342		
Sex, male	0.968 (0.512, 1.833)	0.922			0.824 (0.472, 1.438)	0.495		
BMI	0.986 (0.897, 1.084)	0.986			0.962 (0.891, 1.038)	0.315		
Chronic pain before surgery	1.099 (0.561, 2.151)	0.784			1.019 (0.566, 1.836)	0.949		
Opioids before surgery	1.234 (0.583, 2.612)	0.583			1.158 (0.610, 2.199)	0.654		
Anxiety	0.952 (0.807, 1.122)	0.555			0.939 (0.818, 1.078)	0.373		
Depressed mood	0.920 (0.644, 1.315)	0.648			0.941 (0.714, 1.240)	0.664		
PCS score	1.001 (0.974, 1.028)	0.965			0.993 (0.967, 1.019)	0.590		
Surgery duration	0.998 (0.994, 1.001)	0.145			0.997 (0.995, 1.000)	0.044	0.998 (0.996, 1.001)	0.282
Bleeding	1.000 (0.999,1.000)	0.275			1.000 (1.000, 1.000)	0.113		
Surgery type								
Pelvic resection	**Referent**				**Referent**			
Sacrectomy	1.772 (0.918, 3.421)	0.088	1.577 (0.799, 3.115)	0.189	1.525 (0.847, 2.747)	0.160		
Major complications	2.502 (0.886, 7.066)	0.083	0.442 (0.152, 1.289)	0.135	1.593 (0.744, 3.412)	0.231		

*Abbreviations*: *BMI* Body mass index, *PCS* Pain Catastrophizing Scale, *HR* Hazard ratio

### Factors associated with high vs low pain trajectory groups

We further examined risk factors for pain trajectory group assignment using binary logistic regression (Table 3). Surgery duration and preoperative chronic pain were entered into the final model. A cutoff value for surgery duration was identified as 261 min using the Youden index. A surgery duration longer than 261 min was an independent risk factor for categorization into the high pain group after surgery (odds ratio [OR] 3.754, 95% CI [1.151, 12.240], *p* = 0.028). Moreover, preoperative chronic pain was not independently associated with pain trajectory group assignment (OR 3.332, 95% CI [0.992, 11.194], *p* = 0.052).

**Table 3:** Univariate analysis comparing patient characteristics and surgical factors between the high and low pain groups

	High pain (*n* = 22)	Low pain (*n* = 37)	p
Age (yrs)	46.5 ± 13.0	40.3 ± 12.3	0.074
Sex, male, n (%)	10 (45.5)	20 (54.1)	0.523
BMI (kg/m^2^)	23.8 ± 3.4	23.6 ± 3.8	0.841
Education			0.978
Completed high school, n (%)	13 (59.1)	22 (59.5)	
Less than high school, n (%)	9 (40.9)	15 (40.5)	
Baseline EQ-5D score	0.83 (0.72, 1.00)	0.95 (0.87, 1.00)	0.039
Charlson Comorbidity Index	3 (2, 5)	2 (2, 6)	0.436
Chronic pain before surgery, n (%)	12 (54.5)	8 (21.6)	0.010
Opioids before surgery, n (%)	7 (31.8)	8 (21.6)	0.384
Anxiety	0 (0, 3)	0 (0, 2)	0.340
Depressed mood	0 (0, 0)	0 (0, 0)	0.294
PCS score	8 (2, 26)	0 (0, 13)	0.041
Procedures			0.971
Pelvic resection, n (%)	12 (54.5)	20 (54.1)	
Sacrectomy, n (%)	10 (45.5)	17 (45.9)	
Surgery duration (min)	282 (193, 380)	196 (158, 249)	0.005
Bleeding (ml)	1700 (800, 2500)	900 (600, 1600)	0.014

Data are shown as mean ± SD, median (IQR), or numbers (%). *Abbreviations*: *BMI* Body mass index, *PCS* Pain Catastrophizing Scale

## Discussion

To our knowledge, this is the first study to investigate pain trajectories in patients undergoing pelvic and sacral tumor surgery. Two distinct groups (high vs low pain) of worst and average pain trajectories in the first 2 weeks following surgery were identified. After adjustment, the high pain group of worst pain trajectories was independently associated with prolonged opioid use, indicating relevance to long-term pain outcome. A longer surgical duration was independently associated with assignment to the high pain group, whereas patient-specific characteristics were not.

Different patterns of acute pain trajectories after various surgical procedures have been reported [17, 18]. In our study, we analyzed the pain trajectories of the first 2 weeks after surgery, which is a crucial period during which the patients experienced the most pain. Compared with a traditional one-time pain measurement analysis, a pain trajectory analysis may provide more insight into the time course of pain, including pain persistence and resolution, which may better represent patients’ experiences over the healing process after surgery. In this study, a model of high vs low pain trajectory groups was generated. Compared with the low pain group, the high pain group showed a similar trend but a higher pain level.

We found that the high pain group of worst pain trajectories had delayed pain resolution and opioid cessation. For oncologic surgeries, we should be aware of the possibility of tumor recurrence when patients report persistent pain. Fortunately, no recurrence occurred in this study. Previous studies found that a high pain trajectory increased 30-day readmissions [19] and led to pain persistence and longer opioid use after surgery [20]. Our study adds to existing evidence and highlights the importance of pain control early after surgery. An observational study found that postoperative complications were associated with persistent postsurgical pain [21]; however, this association was not observed in this study. Our results indicated that high pain trajectory was independently associated with prolonged opioid use. Long-term opioid use is a major public crisis [22] and may increase the risk of all-cause mortality [23]. Tracking early pain trajectories can help clinicians identify high-risk patients and provide multidisciplinary pain recourses targeting pain trajectories and reducing opioid prescriptions.

Risk factors associated with categorization into the high pain trajectory group were also investigated. Preoperative opioid use is commonly correlated with postoperative pain outcome, but this correlation was not observed in this study. Similarly, some previous studies also reported no correlation between opioid agents or doses and pain outcomes [17, 24]. One cohort study involving a mixed surgery group reported that patient characteristics, such as age, sex and psychological factors, but not surgical factors, were associated with early pain trajectories [17]. However, in this study, patient factors were not associated with pain outcomes. After multivariable adjustment, only surgical duration was independently associated with pain trajectory group assignment. Orthopedic surgery is associated with the most severe acute pain [25, 26], and pelvic and sacral tumor surgery is the most invasive. It is rational that a longer surgical time indicates more extensive and invasive intervention, thus leading to worse pain outcomes. We speculated that the surgical procedure was a strong risk factor and might weaken the impact of patient factors. Consistently, Tai Y-H et al. found that a longer anesthesia time was related to higher pain intensity and slower pain resolution [27]. Clinicians should be aware of the risk of a high pain trajectory when the surgical duration lasts more than 4 hours and should pay more attention to pain management.

## Strengths and limitations

The key strengths of this study include its prospective design, long-term observation and application of validated questionnaires. The results of repeated pain measurements are superior to previous findings from single measurements at predetermined time intervals (for example, 6 months or 12 months) with regard to reliability [28]. All patients were consecutively included and completed thorough assessments, and the rate of follow-up was high. However, there are several limitations to be discussed.

First, we defined pain resolution as two consecutive reports of no pain without pain control therapies, and follow-ups were terminated when pain resolution was reported. It is not clear whether postsurgical pain recurs months later. Second, we used pain trajectories during the first 2 weeks for acute pain analysis. It is unclear whether earlier postoperative assessment would have similar predictive power. The critical period for the assessment of immediate postoperative pain should be determined in the future. Third, this pilot study involved a small sample size because pelvic and sacral tumors are rare pathologies [29, 30]. These results are preliminary, and larger trials are required to confirm these findings. However, given the strengths of this study, this sample may be representative of this population for determining pain trajectories.

## Conclusions

Postoperative pain is a significant problem among patients undergoing pelvic and sacral tumor surgeries**.** Two distinct high vs low pain trajectory groups were identified, and the high pain trajectory group was associated with delayed recovery after surgery. Longer surgical duration was a risk factor for a high pain trajectory early after surgery. Further studies are necessary to examine pain interventions targeting postoperative pain trajectories and long-term outcomes.

## Data Availability

The datasets used and analyzed during the current study are available from the corresponding author on reasonable request.

## Abbreviations

POD: Postoperative day
PCS: Pain Catastrophizing Scale
SD: Standard deviation
IQR: Interquartile range
HR: Hazard ratio
OR: Odds ratio
BMI: Body mass index
APS-POQ-R: Revised American Pain Society Patient Outcome Questionnaire
EQ-5D-5L: EuroQol five-dimensional-5 levels questionnaire
